# Neuroinflammation and Neurodegeneration: The Promising Protective Role of the Citrus Flavanone Hesperetin

**DOI:** 10.3390/nu12082336

**Published:** 2020-08-05

**Authors:** Egeria Scoditti

**Affiliations:** National Research Council (CNR) Institute of Clinical Physiology (IFC), 73100 Lecce, Italy; egeria.scoditti@ifc.cnr.it; Tel.: +39-0832298860

Over the past 20 years, there has been a remarkable increase in the scientific interest in polyphenols, bioactive compounds naturally present in plant foods and beverages, due to the recognition of their biological actions, their great abundance in the human diet, and their plausible role in the prevention of various chronic degenerative diseases, such as cancer, cardiovascular and neurodegenerative diseases [1]. Great advancements have been made in understanding the health effects of polyphenols in several pathophysiological settings, their mechanism(s) of action, as well as their bioavailability and metabolism, opening up new opportunities for the food and nutraceutical industry.

Over 500 different molecules with a polyphenol structure (i.e., several hydroxyl groups on aromatic rings) with different properties and bioavailabilities have been identified in edible plants, where they are synthetized as secondary metabolites for defense against biotic and abiotic stress. They are divided into five main groups according to structure: phenolic acids, flavonoids, stilbenes, lignans and others. The flavonoid class is further divided into six subclasses, including flavonols, flavones, isoflavones, flavanones, anthocyanidins, and flavanols [2].

Flavonoids are among the most consumed polyphenols: in the European cohort of the PREDIMED (PREvencion con DIeta MEDiterranea) study aimed at assessing the effect of the Mediterranean diet, rich in polyphenols, on the primary prevention of cardiovascular disease, the mean total polyphenol intake was 820 ± 323 mg daily, of which 443 ± 218 mg daily was provided by flavonoids [3], mainly from fruits, vegetables, alcoholic and non-alcoholic beverages, and cereals. In US adults, the estimated mean daily intake of flavonoids was 189.7 ± 11.2 mg, mainly from tea, citrus fruit juices, wine, and citrus fruits [4].

Epidemiological studies found an inverse association between dietary consumption of flavonoids and the risk of cardiovascular diseases, metabolic diseases, neurodegenerative diseases and cancer, with experimental evidence highlighting the contributory role by several flavonoid classes/subclasses and individual compounds to the observed health benefits [5]. Among flavonoids drawing growing scientific attention, there are the flavanones hesperidin (hesperetin-7-O-rutinoside) and its aglycone hesperetin, which are present in high concentrations in citrus fruit and juices, although they can be found in tomatoes and certain aromatic plants, such as mint [6]. Considering the high intake of citrus fruits and juices worldwide, flavanones significantly contribute to the dietary intake of flavonoids [3,4,7]. Hesperidin represents 90% of total flavanones in oranges and orange juices. Orange juice contains between 200 and 600 mg hesperidin/L, and a much higher concentration of flavanones can be found in the whole fruit where the solid parts (the albedo and the membranes) are particularly rich in flavanones [6]. The average daily intake of flavanones in a large cohort of European countries was estimated to be 25.7 ± 27.1 mg, being most often consumed as fruits (72.0%), juices (17.2%), wine (5.4%), and soft drinks (1.7%) [8]. In the Spanish PREDIMED cohort, flavanones were among the most consumed polyphenols, with a daily intake of 132 ± 125, mainly provided by oranges (91%), orange juice (8%), red wine (0.5%), tomatoes (0.1%) [3]. Specifically, the hesperidin daily intake was 90.29 ± 85.89 mg, mostly contributed by the consumption of oranges and their products [3]. In US adults, the total flavanone intake was 14.4 ± 0.6 mg, mostly from fruit and fruit juices, precisely citrus fruit juices [4].

Interestingly, flavanones are among the flavonoid compounds with the highest bioavailability [2]. Once ingested, hesperidin is first metabolized by intestinal bacteria into its aglycone hesperetin, which is more absorbable and bioavailable than hesperidin, and is subsequently transformed into various phenolics and mostly conjugated into glucuronides and sulfates. As such, the biological effects of flavanones, as for polyphenols in general, are likely contributed by the original compounds and mostly their circulating metabolites [9]. After an intake of 130–220 mg hesperetin given as orange juice, the plasma concentrations of hesperetin metabolites have been shown to reach 1.3–2.2 µmol/L [2,10]. 

These interesting data have spurred intensive research on the health-promoting effects of flavanone-rich foods and extracts, and particularly on their most abundant representatives, hesperidin and hesperetin, in cell and animal models, as well as in humans. Preclinical and clinical findings indeed show that hesperidin, hesperetin and their metabolites possess various physiological activities, including antioxidant, anti-inflammatory, hypolipidemic, antihypertensive, insulin-sensitizing, vasculoprotective and anti-atherosclerotic activities, which could explain the beneficial effects of flavanone-rich foods and beverages against cancer, cardiovascular, metabolic and joint arthritis diseases [11,12,13,14]. The antioxidant properties are likely due to both direct radical scavenging activity and an increase in cellular antioxidant defenses via the upregulation of nuclear factor (erythroid-derived 2)-like 2 (Nrf-2) pathway [11]. Furthermore, hesperidin and hesperetin have been reported to inhibit inflammatory responses via the attenuation of the activation of the master pro-inflammatory and redox-sensitive transcription factor nuclear factor (NF)-κB, which induces the expression of cytokines, chemokines, and enzymes with pro-inflammatory action [15,16]. The modulation of the MAPK signaling pathways involved in inflammation, cell survival, apoptosis, and oxidative stress responses also contributes to the anti-inflammatory effects of hesperidin and hesperetin in different pathophysiological settings [17,18]. 

A relatively new field of research regards the protective effects of citrus flavanones on the central nervous system, as first suggested by their antioxidant and anti-inflammatory activities in peripheral tissues coupled with their known ability to cross the blood–brain barrier [2]. This is particularly interesting because neurodegenerative diseases, including Alzheimer’s diseases, Parkinson’s disease, Huntington’s disease, and amyotrophic lateral sclerosis, are debilitating disorders that are increasingly prevalent in modern ageing societies, and are major causes of cognitive and motor dysfunction. Known basic pathogenic pathways in neurodegenerative diseases include neuroinflammation, altered synaptic transmission, deposition of protein aggregates, apoptosis, mitochondrial dysfunction, and oxidative stress [19]. A common feature in neurodegenerative diseases playing a crucial pathophysiological role in their onset and progression is neuroinflammation, which occurs in response to central nervous system trauma, infection, toxins, misfolded and aggregated proteins or as a consequence of autoimmunity, and is mediated by the activation of glial cells, including microglia and astrocytes, that represent the resident innate immune cells in the brain [20]. Once activated through the involvement of MAPK and NF-κB pathways, glial cells increase the production of pro-inflammatory mediators including cytokines, chemokines, adhesion molecules, receptors, and enzymes, and of reactive oxygen species (ROS), as well as nitric oxide (NO), with a consequent unbalance between their neuroprotective and neurotoxic effects and alteration of the cross-talk with neurons, which leads to compromised neuronal functional integrity and further inflammatory response in a chronic vicious manner, resulting in neuronal death and central nervous system injury [20]. Strategies targeting these pathogenic neuroinflammatory processes are currently a rapidly emerging field of research in the search for innovative therapeutic approaches in neurodegenerative diseases. 

Different flavonoids have been demonstrated to exert neuroprotective and anti-inflammatory effects in vitro and in vivo by modulating key cellular processes implicated in neurodegeneration [21]. As previously reviewed [22], citrus flavanones including hesperidin, hesperetin and naringenin exhibit a plethora of interesting protective properties in neurons and glial cells with relevance to central nervous system disorders.

Hesperidin and hesperitin have indeed been found to protect neurons against cytotoxicity induced by oxidative, inflammatory stimuli, and neurotoxic substances, both in vitro and in animal models of neurodegeneration [22]. Hesperidin and hesperetin promote neuron survival through the activation of the pro-survival PI3-Akt and MAPK pathways, and the recruitment of neural progenitor cells via effects on astrocytes [23]. Protective underlying mechanisms may include the antioxidant and anti-inflammatory action of citrus flavanones, as well as improvement of mitochondrial dysfunction and energy metabolism, attenuation of calcium levels, caspase activity, increase in brain-derived neurotrophic factor (BDNF), modulation of apoptotic effector proteins, inhibition of nitrosative stress and the nitrergic pathway [22]. 

In addition, citrus flavanones significantly modulate the inflammatory responses by glial cells. Hesperetin has been demonstrated to decrease the over-expression of inducible NO synthase (iNOS), and pro-inflammatory cytokines (IL-1β, TNF-α and IL-6) as well as the activation of MAPK ERK1/2 and p38, and the transcription factor NF-κB in lipopolysaccharide (LPS)-stimulated BV-2 microglial cells [18]. Moreover, hesperetin significantly suppressed astrocyte and microglial activation in the LPS-challenged mouse brain [18]. 

The antioxidant, anti-inflammatory and signaling pathway-modulating activities of flavanones might contribute to the observed improvement of cognitive and/or motor impairments as previously reported by hesperidin in animal models of Alzheimer’s disease, Parkinson’s disease, Huntington’s disease and epilepsy [24], and by hesperetin in animal models of Alzheimer’s diseases [25], Parkinson’s disease [26], and epilepsy [27]. Furthermore, benefits in psychiatric disorders exerted by hesperidin and hesperetin have been previously suggested through their anti-depressant and anxiolytic effects, as documented in animal models [22].

In line with these findings and expanding the neuropharmacological properties of the less studied but more bioavailable flavanone hesperetin, a preclinical study published in *Nutrients* addressed the neuroprotective role and potential mechanisms of hesperetin against LPS-induced Toll like receptor (TLR)-4-mediated neuroinflammation and related neurodegeneration [28]. In particular, Muhammad et al. pretreated mice with hesperetin (50 mg/kg daily) for three weeks before LPS challenge for further two weeks, inducing neurodegenerative features. They monitored markers of neuroinflammation, neuronal apoptosis, and oxidative stress, as well as memory impairments in the brain, and confirmed some results in vitro in LPS-stimulated neuronal and microglial cell lines cotreated with 50 µmol/L hesperetin. Hesperetin was found to significantly rescue LPS-induced activation of microglia (gliosis) and astrocytes (astrocytosis) in cortical and hippocampal regions of mouse brain, and the corresponding proinflammatory activation as indexed by the reduced activation of NF-κB and expression of the cytokines IL-1β and TNF-α in the mouse brain and BV-2 microglial cells. Concordantly, hesperetin overcame the overproduction of ROS and the reduction in antioxidant proteins such as Nrf2 and heme-oxygenase (HO)-1 stimulated by LPS in mouse brain. This was further supported by cytoprotective effects against LPS-induced cytotoxicity and oxidative stress in the mouse hippocampal neuronal HT-22 cell line. Accordingly, hesperetin was able to prevent LPS-induced neuronal apoptosis and neuronal loss in vivo [28]. 

In agreement with these beneficial effects against neuroinflammation, apoptosis and oxidative stress, critical processes in neurodegeneration, hesperetin has been found to improve synaptic dysfunction as well as memory, learning and cognitive impairments following LPS stimulation. Besides the known increase in BDNF level in the hippocampus that could mediate the hesperetin-induced memory-enhancing effect, the authors demonstrated that these effects might also be mediated by the recovery of the activation of cAMP response element binding protein (CREB) [28], a transcription factor implicated in memory formation, as observed in the cortex and hippocampus of treated mice. 

A previous study by the same group [29] showed similar findings in an animal model of Alzheimer’s disease-like neurodegeneration. Indeed, hesperetin counterbalanced abnormal ROS production, activation of astrocytes and microglia, apoptotic cell death, neuroinflammation, and synaptic dysfunction in an in vivo amyloid β (Aβ)-injected mouse model of Alzheimer’s disease, and confirmed in vitro in Aβ-treated neuronal and microglial cells. Improvements in markers of Aβ pathology in the mice brain and in cultured neuronal cells, as well as in memory dysfunction in response to Aβ injection, were also here documented for hesperetin [29]. Therefore, a common underlying anti-neuroinflammatory and antioxidant action by hesperetin may be suggested as a mechanism preventing neurodegeneration in response to different agents detrimental to the central nervous system. 

Collectively, this evidence in animals and in vitro lays the foundation for novel perspectives in the prevention of neurodegeneration, and contributes to explaining earlier human evidence from observational and interventional studies indicating improved cognitive function and lowered risk of dementia with frequent consumption of flavanone-rich citrus fruits or juices. Indeed, in a randomized controlled human intervention trial, 8 week daily consumption of 500 mL flavanone-rich (305 mg with 549 mg hesperidin/L) orange juice improved cognitive function compared with the consumption of a flavanone-low (37 mg with 64 mg hesperidin/L) orange juice in healthy older adults [30]. Similarly, the consumption of 240 mL of orange juice containing 220.46 mg hesperidin acutely enhanced cognitive performance in healthy middle-aged men [31], and citrus consumption was inversely and dose-dependently correlated with the risk of incident dementia in a cohort of elderly subjects [32]. 

Translation of preclinical data to humans may be challenging, and further investigations are required on the flavanone molecular targets and bioactive metabolites, doses, length of treatment, patient population, and interindividual variability in biological responses, as well as the interaction with drugs. The work of Muhammad et al. [28] provided novel mechanistic insights into the neuroprotective effects of hesperetin, and called for future flavanone-based dietary intervention studies in humans to substantiate them as new potential agents contributing to the prevention and/or treatment of neurodegenerative disorders. 
